# Reduced expression of microRNA-130a promotes endothelial cell senescence and age-dependent impairment of neovascularization

**DOI:** 10.18632/aging.103340

**Published:** 2020-05-26

**Authors:** Wahiba Dhahri, Sylvie Dussault, Éliane Légaré, François Rivard, Michel Desjarlais, Raphael Mathieu, Alain Rivard

**Affiliations:** 1 Department of Medicine, Centre Hospitalier de l’Université de Montréal (CHUM), Research Center Montréal, Montréal, Québec Canada

**Keywords:** angiogenesis, aging, senescence, microRNA, neovascularization

## Abstract

Aging is associated with impaired neovascularization in response to ischemia. MicroRNAs are small noncoding RNAs emerging as key regulators of physiological and pathological processes. Here we investigated the potential role of microRNAs in endothelial cell senescence and age-dependent impairment of neovascularization. Next generation sequencing and qRT-PCR analyses identified miR-130a as a pro-angiogenic microRNA which expression is significantly reduced in old mouse aortic endothelial cells (ECs). Transfection of young ECs with a miR-130a inhibitor leads to accelerated senescence and reduced angiogenic functions. Conversely, forced expression of miR-130a in old ECs reduces senescence and improves angiogenesis. In a mouse model of hindlimb ischemia, intramuscular injection of miR-130a mimic in older mice restores blood flow recovery and vascular densities in ischemic muscles, improves mobility and reduces tissue damage. miR-130a directly targets antiangiogenic homeobox genes MEOX2 and HOXA5. MEOX2 and HOXA5 are significantly increased in the ischemic muscles of aging mice, but forced expression of miR-130a reduces the expression of these factors. miR-130a treatment after ischemia is also associated with increased number and improved functional activities of pro-angiogenic cells (PACs). Forced expression of miR-130a could constitute a novel strategy to improve blood flow recovery and reduce ischemia in older patients with ischemic vascular diseases.

## INTRODUCTION

Aging is an important risk factor for atherosclerotic cardiovascular diseases. Advanced atherosclerosis in the elderly is likely to manifest irreversible changes, including severe and diffuse obstructive lesions leading to impaired tissue perfusion. In that situation, the capacity of the organism to grow new blood vessels (neovascularization) represents an important adaptive mechanism to prevent ischemia [1]. Neovascularization is classically linked to angiogenesis, which is defined as the proliferation and migration of mature endothelial cells leading to extension of a pre-existing vascular network [2]. Besides angiogenesis, postnatal neovascularization is also regulated by the activities of bone marrow-derived pro-angiogenic cells (PACs) [3, 4]. PACs are incorporated into ischemic tissues where they stimulate neovascularization mainly through paracrine secretion of growth factors and cytokines [5].

Unfortunately, in addition to the increased risk associated with atherosclerotic vascular diseases, another consequence of advanced age is an impairment of defense mechanisms against different stresses, including ischemia. For example, aging is associated with impaired neovessel development after arterial occlusion in several animal models [6, 7]. In addition, the number and the angiogenic activities of PACs have been found to be impaired by aging in animals and humans [8–11]. However, the exact mechanisms leading to reduced neovascularisation and PAC function with advanced age remain to be determined.

MicroRNAs (miRNAs or miRs) are a novel class of endogenous non-coding small RNA molecules (20-25 nucleotides) that regulate several physiological and pathological processes [12, 13]. Although miRNAs are appreciated as important regulators of cell senescence and age-associated diseases such as cancers [14], their specific role for the modulation of vascular function during aging remains to be determined. The key role of miRNAs in angiogenesis and endothelial cell function was previously revealed by disrupting Dicer and Drosha, two enzymes involved in miRNA biogenesis [15, 16]. Several miRNAs have since been found to promote angiogenesis in different context, and these miRs have collectively been referred to as pro-angiomiRs [12, 13, 17]. Here we hypothesized that reduced expression of pro-angiomiR(s) could contribute to impair vascular function and neovascularization in the context of aging. The present study shows for the first time that reduced expression of miR-130a contributes to age-dependent endothelial cell senescence, and that this is associated with impairment of angiogenesis, PAC function and ischemia-induced neovascularization. We propose that forced expression of miR-130a could represent a novel therapeutic strategy to reduce ischemia in older patients with severe vascular diseases.

## RESULTS

### Effect of aging on miRNA expression

**Figure 1:**
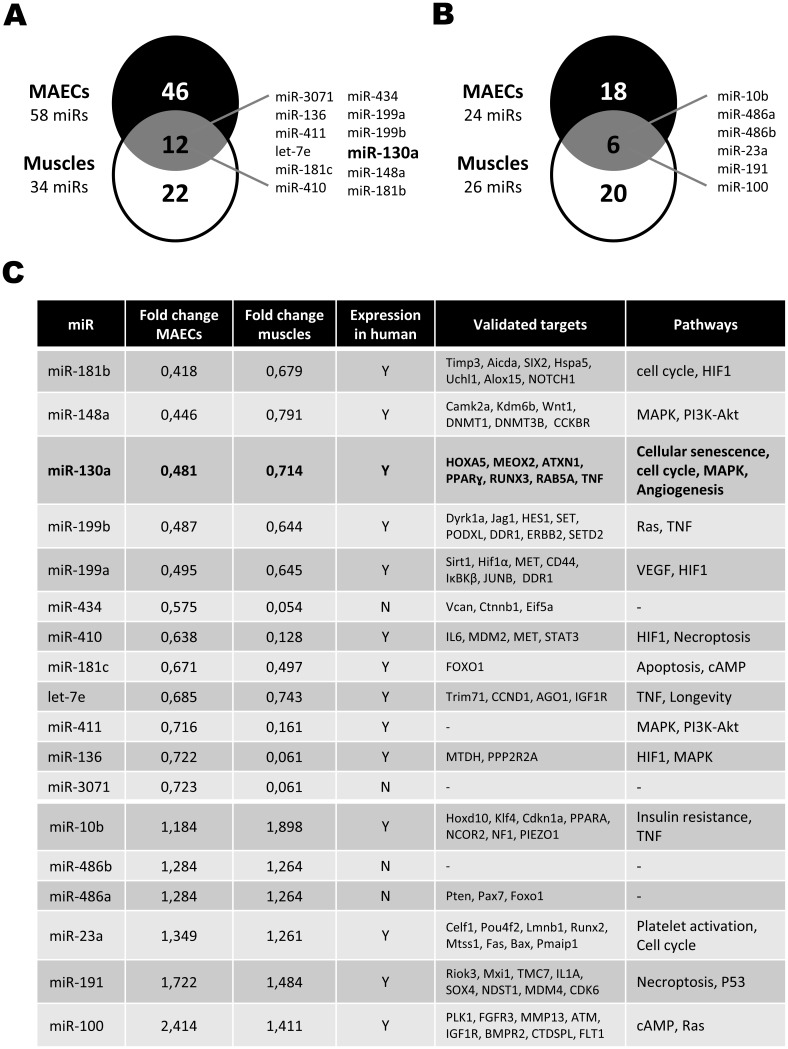
**Effect of aging on the modulation of microRNA expression.** (**A**, **B**) Venn diagrams showing the number of miRNAs that are downregulated (**A**) or upregulated (**B**) in Mouse Aortic Endothelial Cells (MAECs) and ischemic muscles from old vs. young mice, as assessed by next-generation sequencing (pool of 3-4 mice/group). miRNAs modulated both in MAECs and ischemic muscles are listed. (**C**) List of miRNAs modulated by at least 15% in old animals both in MAECs and ischemic muscles. Validated targets and potential pathways involved are shown for each miRNA.

Next generation sequencing (NGS) was used to evaluate the expression of miRNAs in endothelial cells isolated from the aorta of young (6-8 weeks) and old (15-24 months) C57Bl6 mice. In parallel experiments, NGS was also used to compare miRNA expression in ischemic hindlimb muscles of young and old mice. miRNAs with at least 250 reads per million reads mapped-(RPM) and modulated by 15% or more were included in the analysis. In endothelial cells, aging resulted in more miRNAs being downregulated (Figure 1A) compared to miRNAs that were upregulated (Figure 1B). Among 58 miRNAs that were reduced in old endothelial cells, 12 were also found to be reduced in the ischemic muscles of aging mice (Figure 1A). By comparison, only 6 miRNAs were found to be upregulated both in endothelial cells and muscles of aging mice, including 2 miRNAs (miR486a and miR486b) that are not found in humans (Figure 1C). Interestingly miR-130a, one of the most downregulated miRNAs in aging mice, is predicted to modulate pathways involved in the modulation of both angiogenesis and cellular senescence (Figure 1C). Therefore, in the following experiments, we focused on characterizing the role of miR-130a in the modulation of endothelial cell senescence and angiogenesis.

### miR-130a modulates endothelial cell senescence and angiogenic activities

**Figure 2:**
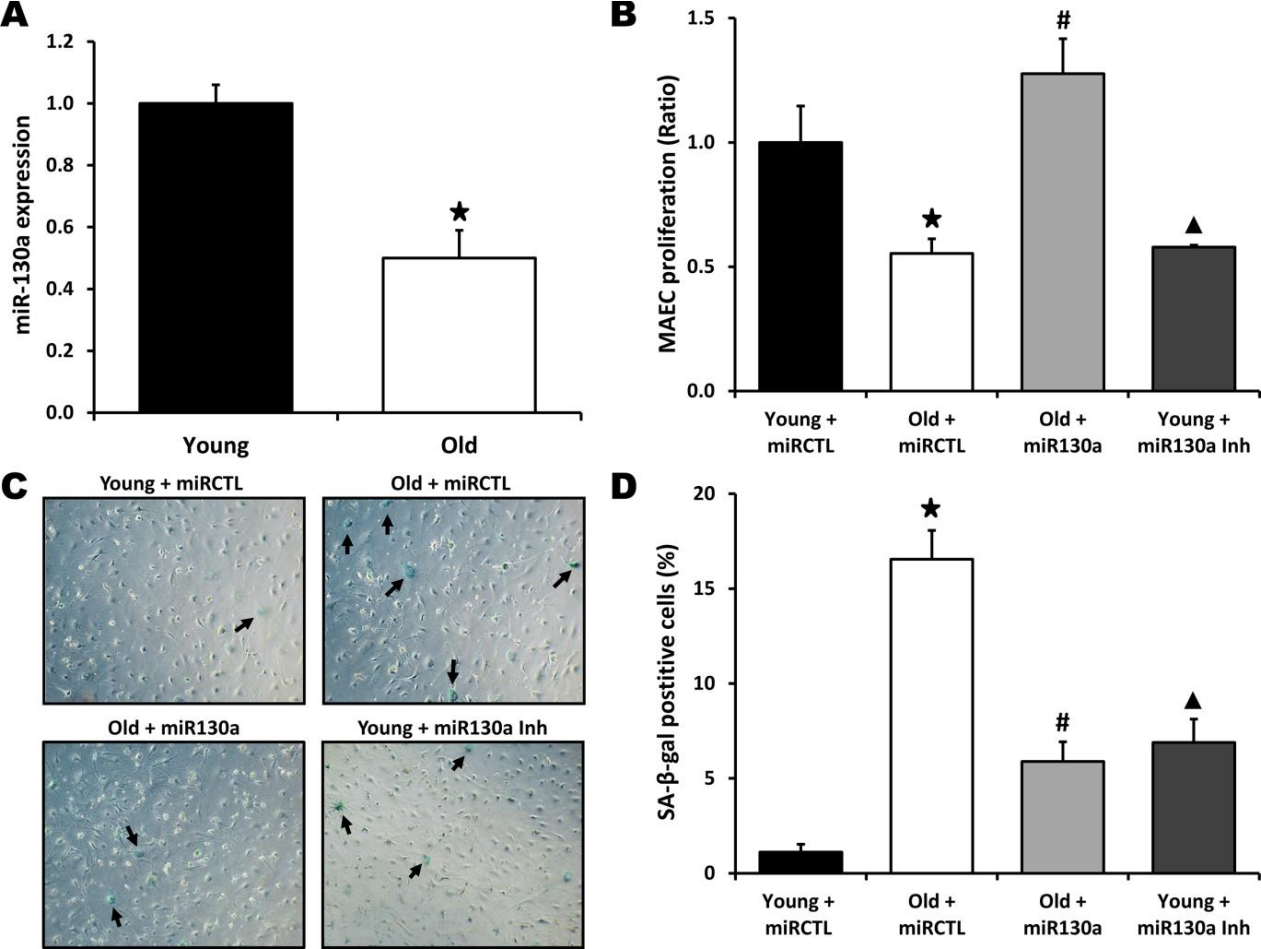
**miR-130a and endothelial cell senescence.** (**A**) Relative expression of miR-130a in Mouse Aortic Endothelial Cells (MAECs) isolated from young and old mice, as quantified by real-time qPCR (n=3/group). (**B**–**D**) Evaluation of cell proliferation (MTS assay, **B**) and senescence (Senescence-associated β-Galactosidase staining, **C**, **D**) in MAECs isolated from young or old mice and treated with miR-130a mimic (miR-130a), anti-miR-130a (miR-130a inh), or a scrambled miR mimic control (miRCTL). Data are mean ± SEM (n=3/group). * p<0.05 vs. young+miRCTL; # p<0.05 vs. old+miRCTL. ▲ p<0.05 vs. young+miRCTL.

**Figure 3:**
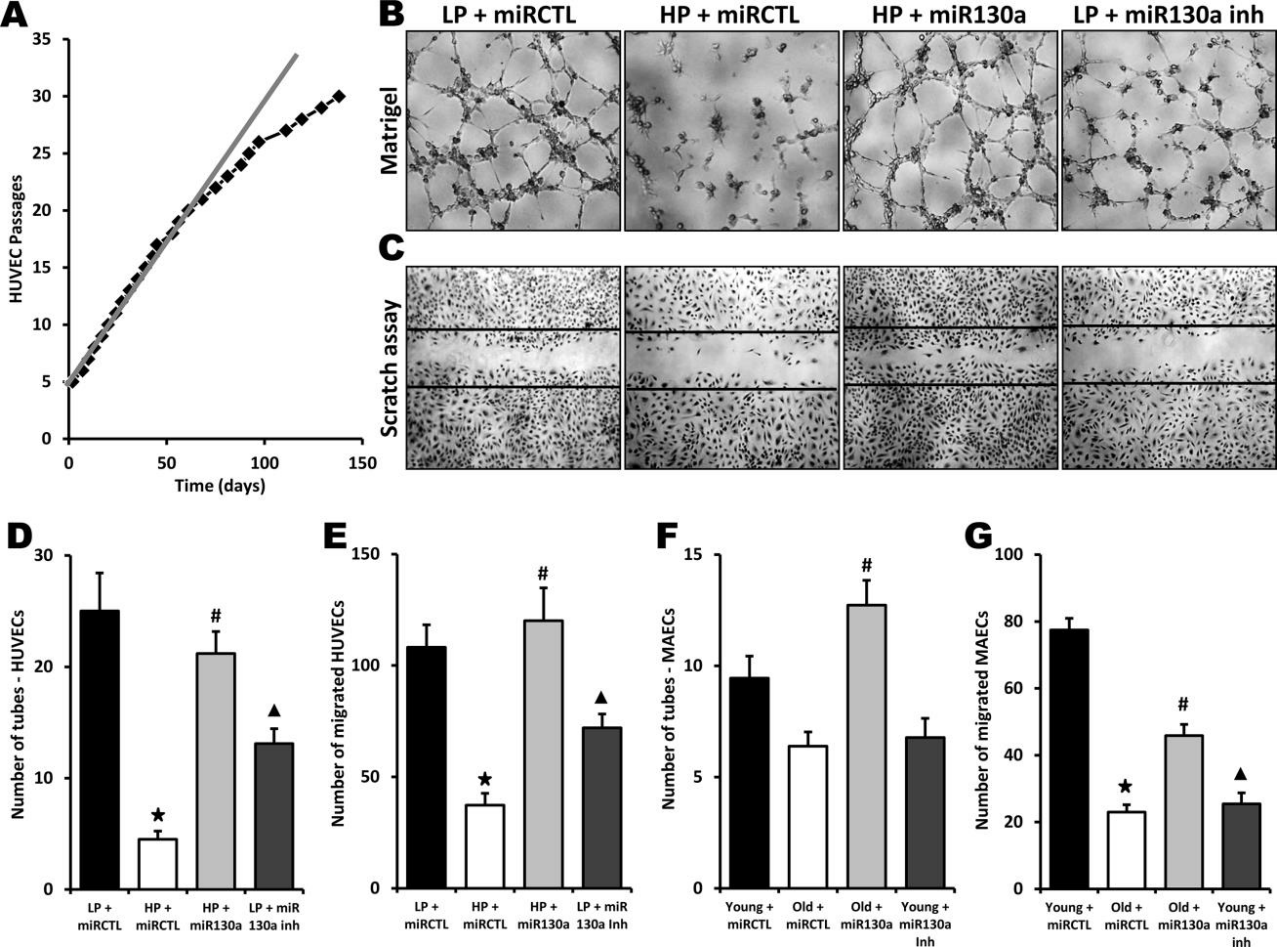
**Role of miR-130a in age-dependent impairment of endothelial cell angiogenic activities.** (**A**) Graph showing serial passaging over time in HUVECs. An important reduction of cell proliferation is seen after 25 passages. Low-passage HUVECs (LP, 4-6 passages) were compared to high-passage HUVECs (HP, 28 passages or more) in the following experiments. (**B**–**E**) Evaluation of angiogenesis and cell migration in vitro using matrigel (**B**, **D**) and scratch (**C**, **E**) assays in LP or HP HUVECs treated with miR-130a mimic (miR-130a), anti-miR-130a (miR-130a inh), or a scrambled miR mimic control (miRCTL). Data are mean ± SEM (n=5/group). * p<0.05 vs. LP+miRCTL; # p<0.05 vs. HP+miRCTL. **▲** p<0.05 vs. LP+miRCTL. (**F**, **G**) Quantification of angiogenesis and cell migration in vitro using matrigel (**F**) and scratch (**G**) assays in MAECs isolated from young or old mice and treated with miR-130a mimic (miR-130a), anti-miR-130a (miR-130a inh), or a scrambled miR mimic control (miRCTL). Data are mean ± SEM (n=3/group). * p<0.05 vs. young+miRCTL; # p<0.05 vs. old+miRCTL. **▲** p<0.05 vs. young+miRCTL.

To investigate the role of miR-130a in vitro, we used gain- and loss-of-function approaches in endothelial cells isolated from young or old mice. Mouse aortic endothelial cells (MAECs) were transfected with a miR-130a mimic or an anti-miR-130a. Using RT-PCR, we first confirmed that miR-130a is significantly reduced (50% reduction) in old vs. young endothelial cells (Figure 2A). We found that cellular proliferation was significantly reduced (45% reduction, Figure 2B) and cell senescence increased (15 fold increase, Figure 2C, 2D) in MAECs isolated from old vs. young mice. However, transfection with miR-130a mimic reduces cell senescence by 64% and restores the proliferative activity of old MAECs (Figure 2B–2D). Conversely, transfection of young ECs with a miR-130a inhibitor leads to accelerated senescence (6.3 fold increase) and reduces EC proliferative activity by 42% (Figure 2B–2D). We then used 2 different in vitro models to confirm the potential role of miR-130a on the angiogenic functions of endothelial cells in the context of aging: 1) MAECs isolated from young vs. old mice and 2) human umbilical vein endothelial cells (HUVECS) that were used at low (4-6) passages (young) or high (>28) passages (old) with reduced proliferative activity (Figure 3A). In both models aging was associated with impaired tube formation (82% and 32% reduction respectively) and cellular migration (66% and 70% reduction respectively), and these functional activities could be at least partly restored following transfection with miR-130a mimic (Figure 3B–3G). On the other hand, inhibition of miR-130a impaired tube formation (48% and 28% reduction) and cell migration (33% and 67% reduction) in young endothelial cells (Figure 3B–3G).

### miR-130a treatment rescues age-dependent impairment of neovascularization

**Figure 4:**
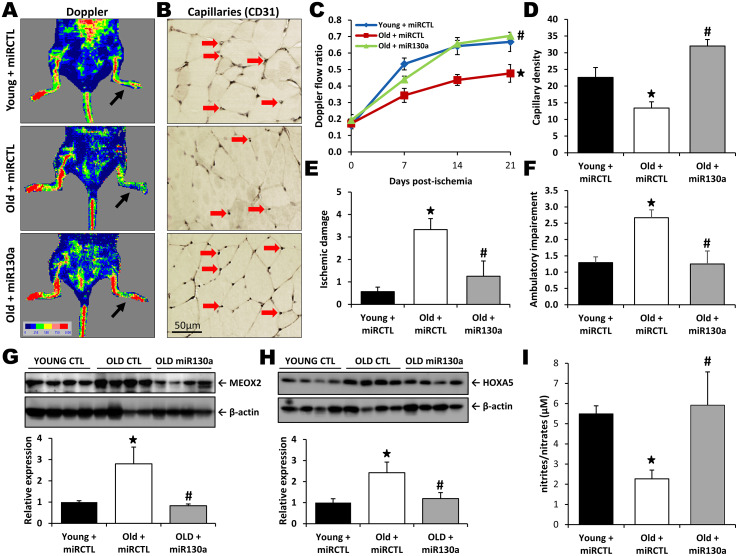
**miR-130a treatment rescues age-dependent impairment of neovascularization.** Quantification of blood flow recovery (Laser Doppler, **A**, **C**), capillary density (CD31 immunostaining, **B**, **D**), ischemic damage (**E**) and ambulatory impairment (**F**) in young mice treated with a scrambled miR mimic control (miRCTL) or old mice treated with miR-130a mimic (miR-130a) or miRCTL. Arrows indicate left ischemic hindlimbs (**A**) and capillaries (**B**). (**G**, **H**) Representative Western blots and quantitative analyses of MEOX2 (**G**) and HOXA5 (**H**) expression in the ischemic muscles of the three groups of mice. (**I**) Comparison of NO blood plasma bioavailability in the three groups of mice. Data are mean ± SEM (n=3-7/group). * p<0.05 vs. young+miRCTL; # p<0.05 vs. old+miRCTL.

We have previously demonstrated that neovascularization and blood flow recuperation following ischemia are impaired with aging [6]. To study the potential therapeutic effect of miR-130a in vivo, hindlimb ischemia was surgically-induced in old and young mice and blood flow perfusion was assessed by Laser Doppler perfusion imaging (LDPI). As shown on Figure 4A, 4C, blood flow recovery was significantly impaired in old vs. young mice at day 21 after surgery (Doppler flow ratio (DFR) 0.48±0.05 vs. 0.67±0.06; p<0.05). However, old mice treated intramuscularly with a miR-130a mimic demonstrated improvement of blood flow recuperation compared to old mice treated with a scrambled miR mimic control (DFR 0.70±0.02 vs. 0.48±0.05; p<0.05). A similar effect was also observed in microvessels, where aging was associated with a 40% reduction of capillary density whereas intramuscular injection with miR-130a mimic restored vessel densities in the ischemic muscles of old mice (Figure 4B, 4D). Clinically, these effects correlated with reduced ischemic damages (ischemic score 1.25 vs. 3.33, Figure 4E) and improved mobility (ambulatory impairment score 1.25 vs. 2.67, Figure 4F) in old mice treated with miR-130a mimic. miR-130a has previously been shown to directly target and induce post-transcriptional repression of the antiangiogenic homeobox genes MEOX2 (GAX) and HOXA5 [18]. We found that MEOX2 and HOXA5 expression are significantly increased (2.8 fold and 2.4 fold increase, respectively) in the ischemic muscles of old mice compared to young mice. However, treatment of old mice with miR-130a mimic can reduce the expression of these antiangiogenic factors (Figure 4G, 4H). Nitric oxide (NO) is essential for the maintenance of endothelial function and has also been shown to be important for postnatal neovascularization [19]. Here we found that NO bioavailability is reduced by 59% in the blood plasma of old vs. young mice, but that its expression is rescued in old mice that are treated with miR-130a (Figure 4I).

### Effect of miR-130a treatment on the number and the angiogenic activities of PACs in the context of aging

**Figure 5:**
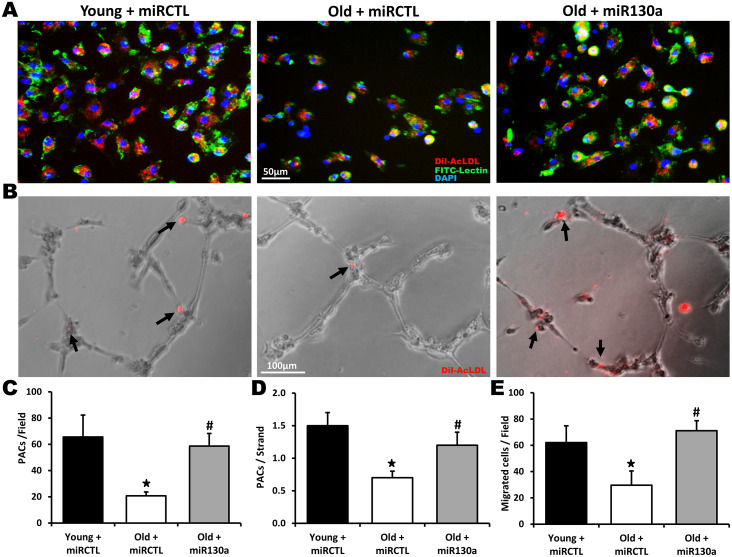
**Effect of miR-130a treatment on PAC number and function during aging.** Representative pictures and quantification of PAC number (**A**, **C**), PAC (red) integration into HUVEC tubules (matrigel assay, **B**, **D**) and PAC migration (Boyden chamber assay, **E**) in young mice treated with a scrambled miR mimic control (miRCTL) or old mice treated with miR-130a mimic (miR-130a) or miRCTL. Triple-stained PACs (DAPI, BS-1 lectin-FITC, and DiI-acLDL) are shown in (**A**). Data are mean ± SEM (n=4-5/group). * p<0.05 vs. young+miRCTL; # p<0.05 vs. old+miRCTL.

Bone marrow-derived PACs can reach ischemic tissues and contribute to the formation of new blood vessels [5]. However, the number and the angiogenic activities of PACs are impaired in older patients with atherosclerotic diseases [9–11]. Here we found that the number of PACs is significantly reduced (68% reduction) in old compared to young animals (Figure 5A, 5C). Moreover the angiogenic activities of PACs including integration into endothelial cell tubules (53% reduction, Figure 5B, 5D) and migration (52% reduction, Figure 5E) were all impaired in older mice. However, treatment with miR-130a mimic restored the number and the angiogenic activities of PACs in older mice (Figure 5A–5E).

## DISCUSSION

To our knowledge, this is the first report demonstrating the important role of a microRNA (miR-130a) for the modulation of both endothelial cell senescence and the physiological response to tissue ischemia in the context of aging. An essential process after ischemia is the ability of the organism to develop new blood vessels (neovascularization) in order to maintain tissue perfusion [1]. In patients with severe ischemic vascular diseases, improving the natural capacity to form neovessels through therapeutic neovascularization would be an attractive strategy to increase blood flow perfusion and avoid tissue damage [1, 20]. Unfortunately, one of the main effect of advanced age is a decline in the capacity to respond to different stresses, including ischemia. For example, aging is associated with defective neovessel formation after arterial occlusion in different animal models [6, 7]. Another example is patients suffering from acute myocardial infarction, in which aging has been associated with reduced collateral artery development [21]. Therefore, despite positive results obtained in classical pre-clinical angiogenesis models using young animals, augmenting neovascularization in aged patients presenting ischemic vascular diseases might prove challenging [22]. Here we identified a novel mechanism involving age-dependent dysregulation of an angiomiR (miR-130a), which is associated with endothelial senescence and impaired ischemia-induced neovascularization. We also demonstrate for the first time the potential beneficial effect of a miRNA mimic supplementation therapy in order to improve endothelial cell function and rescue impaired neovascularization in the context of aging.

We found that miR-130a was one of the most downregulated miRNAs in aging mice, both in ischemic muscles and in endothelial cells isolated from the aorta (MAECs). Interestingly, miR-130a was previously identified as one of nine miRNAs significantly reduced in the peripheral blood mononuclear cells of older individuals [23]. Here we show that reduced miR-130a expression is associated with increased cell senescence in MAECs isolated from old vs. young mice. Using transfection with miR-130a mimic, we could reduce cell senescence in old MAECs. On the other hand, transfection of young MAECs with a miR-130a inhibitor increased cell senescence. This constitutes the first demonstration of the important role of miR-130a for the modulation of endothelial cell senescence. Our results also indicate that miR-130a overexpression increases proliferation, migration and angiogenesis (tube formation) in aging endothelial cells and that conversely, inhibiting miR-130a impairs these functional activities in young endothelial cells. miR-130a has previously been shown to improve survival and promote cellular proliferation in cancer cells [24, 25]. Although less extensively studied in the cardiovascular system, miR-130a was shown to enhance hypoxia-induced smooth muscle cell proliferation and might be involved in the in the development of pulmonary hypertension [26]. On the other hand, miR-130a supplementation with a lentivirus was shown to attenuate cardiac dysfunction and remodeling after myocardial infarction [27]. Interestingly, miR-130a treatment was also associated with increased microvascular density in the myocardium, suggesting that improved neovascularization of ischemic tissues might have contributed to the beneficial effect of miR-130a in that situation [27]. Although pro-angiogenic effects of miR-130a have previously been suggested in the context of cancer [18, 28], the role of miR-130a to promote physiological neovascularization in response to tissue ischemia is unknown. Here, using a well described animal model of hindlimb ischemia, we show that miR-130a supplementation could have important angiogenic effects in ischemic muscles. Aging mice injected with miR-130a demonstrated significant improvement of blood flow perfusion, increased mobility and reduced ischemic damages compared to controls. At the level of microvessels, this was associated with increased capillary density in the ischemic muscles of animals treated with miR-130a. These results represent the first demonstration of the potential therapeutic effect of miR-130a on angiogenesis and neovascularization in the context of aging. Our study was not designed to look at collateral growth (arteriogenesis), which also contributes to neovascularization and blood flow recuperation after ischemia. Future studies are therefore needed to determine the full impact of miR-130a on neovascularization.

The mechanisms by which miR-130a modulates angiogenesis and ischemia-induced neovascularization in aged animals are potentially diverse. The angiogenic effect of miR-130a in vitro has previously been linked to the antiangiogenic homeobox genes MEOX2 (also known as GAX) and HOXA5 [18]. miR-130a was shown to directly target and reduce the expression of both MEOX2 and HOXA5. MEOX2 is expressed at its highest level in quiescent ECs and is down-regulated when ECs are exposed to mitogens, angiogenic signals, or pro-inflammatory factors [18]. MEOX2 has been shown to induce endothelial cell senescence and inhibit angiogenesis in both in vitro and in vivo models [29–31]. It is therefore considered to be a key regulator of EC phenotype in response to proangiogenic and antiangiogenic signals [18]. HOXA5, the other homeobox gene targeted by miR-130a, leads to down regulation of many proangiogenic genes and was shown to block angiogenesis in vitro [32]. In the current study we found that reduced miR-130a expression was associated with increased levels of MEOX2 and HOXA5 in the ischemic muscles of old mice compared to young mice. Therapeutic neovascularization induced by miR-130a mimic in old mice was associated with reduced expression of both MEOX2 and HOXA5 in ischemic muscles. Our results therefore suggest that lower expression of miR-130a with aging leads to increased levels of MEOX2 and HOXA5, accelerated endothelial cell senescence and a failure to adopt an angiogenic phenotype in response to angiogenic signals, which ultimately results in defective ischemia-induced neovascularization. Interestingly, MEOX2 has been shown to repress endothelial nitric oxide synthase (eNOS) expression at the mRNA and protein levels [31]. Since eNOS is an essential mediator of EC migration, VEGF-induced angiogenesis and ischemia-induced neovascularization [19, 33], reduced expression of eNOS due to higher levels of MEOX2 could also contribute to impair neovascularization with aging. This would be consistent with the reduction of NO bioavailability that we documented in the blood plasma of old animals, and the rescue of NO blood levels in old mice that were treated with miR-130a.

The present study also suggests that PACs could be involved in the modulation of neovascularization by miR-130a in the context of aging. PACs can reach ischemic muscles where they stimulate neovascularization either directly by integrating new vessels, or more often indirectly through secretion of angiogenic factors and cytokines [5]. The number and/or the angiogenic activities of EPCs are impaired by aging both in animal models and in humans [8–11]. However, the exact mechanisms that are involved in the reduction of PAC number and function in the context of aging remain to be determined. Here we found that miR-130a supplementation after surgically-induced hindlimb ischemia can improve the number and the angiogenic activities of PACs (migration, integration into tubules) in aging mice. miRNAs are known to be important regulators of progenitor and stem cells in the cardiovascular system [34]. The stimulating effect of miR-130a on PACs in this study is consistent with previous reports that demonstrated the importance of miR-130a to maintain normal autophagy levels, promote PAC survival and preserve normal PAC function [35, 36]. The process by which miR-130a modulates PACs is currently unknown. miR-130a could be released in the circulation and directly influence PACs in the bone marrow or, alternatively, PACs could respond to growth factors secreted by muscles in mice treated with miR-130a. In addition, the specific mechanisms involved in the modulation of PAC functions by miR-130a and whether the same targets (i.e. MEOX2 and HOXA5) are involved remain to be determined.

In conclusion, our study demonstrates for the first time that reduced miR-130a expression promotes endothelial cell senescence and contributes to the impairment of angiogenesis and ischemia-induced neovascularization in the context of aging. Age-dependent reduction of miR-130a expression is associated with induction of the antiangiogenic homeobox genes MEOX2 and HOXA5, together with a reduction of NO bioavailability and impairment of PAC number and function. Our results suggest that forced expression of miR-130a using a miRNA mimic could represent a novel therapeutic strategy to increase blood flow recovery and reduce tissue ischemia in older patients with severe ischemic vascular diseases.

## MATERIALS AND METHODS

### Mouse aortic endothelial cell (MAEC) isolation and culture

Endothelial cells were isolated from the thoracic aorta by an explant technique. The thoracic aorta was gently excised, opened longitudinally and cut into 2mm-long segments. The aortic segments of 2 mice were placed on Matrigel (Basement membrane, BD Biosciences) in a 6-well plate and incubated in DMEM low-glucose supplemented with 10% FBS, 10% Newborn Calf serum, 1% penicillin-streptomycin, 1% fungizone (Life Technologies, Waltham, MA), 90 ug/mL heparin (Sandoz, Boucherville, Qc, Canada) and 50 ug/ml endothelial cell growth supplements (Alfa Aesar, Haverhill, MA) at 37°C in a 95% air/5% CO^2^ incubator. The vessel segments were removed after 7 days in culture. After 10 days, the cells were detached with dispase (BD Biosciences) and then plated onto one 25 cm^2^ flask coated with 1% gelatin. The subsequent passage was performed with 0.25% trypsin-EDTA, and cells were split in a 1:4 ratio. Cells were used at passage 2.

### HUVECs culture

Human umbilical vein endothelial cells (HUVECs) were purchased from Life Technologies (Carlsbad, CA) and cultured in medium 200 (Life technologies) supplemented with 8% foetal bovine serum (FBS, Wisent, St-Jean-Baptiste, QC, Canada), 100 IU/ml penicillin/0.1 mg/ml streptomycin (Wisent) and low serum growth supplement (LSGS; 2% FBS, 3 ng/ml bFGF, 10 mg/ml heparin, 1 mg/ml hydrocortisone, and 10 ng/ml EGF; Life Technologies). Cells were grown at 37 °C, 5% CO_2_ and 95% air, and the medium was changed every 2 days. HUVECs were passaged (1:4) when they reached 90% confluence. For low passage cells, HUVECs were used at passages 4-6. High passage cells (28 passages or more) were generated by passaging successively the cells until changes in morphology and reduced proliferation rates were observed.

### RNA isolation and next generation sequencing analyses

RNA was extracted from endothelial cells and ischemic hindlimb muscles (1 day after surgery) using the Ambion mirVana™ miRNA isolation kit (Life Technologies) according to the manufacturer’s protocol. Quantification of total RNA was made with a nanodrop and 1 ug of total RNA was used for library preparation. Quality of total RNA was assessed with the BioAnalyzer Nano (Agilent) and all samples had a RIN above 8. Library preparation was done with the Truseq Small RNA library preparation kit (Illumina, San Diego, CA, Cat #RS-200-0012). 11 PCR cycles were required to amplify libraries. Libraries were quantified with a nanodrop and the quality was assessed with the BioAnalyzer High Sensitivity (Agilent, Santa Clara, CA). All libraries were diluted to 10 nM, normalized and pooled to equimolar concentration based on Miseq v2 50 cycles using 7 pM of pooled library. Sequencing was performed with the Illumina Hiseq2000 using the Hiseq Reagent Kit v3 (200 cycles, paired-end) and 1.7 nM of the pooled library. Around 70M paired-end reads was generated per sample. Quantification included the raw read count, as well as normalized expression level as RPM (reads per million reads mapped) values to account for the variability in the library size. miRTarBase (http://mirtarbase.mbc.nctu.edu.tw) and miR+Pathway (http://www.insect-genome.com/miR-pathway) databases were used to identify validated targets and determine signalling pathways modulated by specific miRNAs.

### qRT-PCR evaluation of miRNA expression

50 ng of total RNA was reverse transcribed using the TaqMan® MicroRNA Reverse Transcription Kit (Life Technologies) as described by the manufacturer. Before use, RT samples were diluted 1:5. Gene expression level was determined using Taqman MicroRNA assays (Cat #4427975, Life Technologies). qPCR reactions were performed using 1-5 ng of cDNA samples, using Perfecta qPCR Fastmix II (Quanta) and 2 μM of miR-130a primer. The Viia7 qPCR instrument (Life Technologies) was used to detect the amplification level and was programmed with an initial step of 20 sec at 95°C, followed by 40 cycles of: 1 sec at 95°C and 20 sec at 60°C. Relative expression (RQ = 2^-ΔΔCT^) was calculated using the Expression Suite software (Life Technologies), and normalization was done using both U6 snRNA and SnoRNA 202.

### miRNA transfection in MAECs and HUVECs

Transfections were carried out at a concentration of 50 nM using Lipofectamine RNAiMAX Reagent (Life Technologies) according to the manufacturer’s protocol and as previously described [37]. HUVECs were transfected 24 hours after being plated in 6-well plates with the following miRs (Life Technologies): miRVana miR mimic negative control #1, miRVana miR mimic miR130a, miRVana anti-miR negative control #1, miRVana anti-miR miR130a inhibitor. After 24 hours, the transfection medium was replaced with antibiotic-free complete medium. Transfection efficiency was measured using mimic transfection control Dy547 (Dharmacon) and found to be 80-90%.

### Proliferation assay

Cell proliferation was assessed using the MTS Celltiter 96 aqueous non-radioactive cell proliferation assay (Promega, Madison, WI, USA). 15 000 cells were plated in 96-well plates coated with 1% gelatin. Once cells were attached, they were incubated for 24 hrs with 50 ng/ml of VEGF. After the treatments, MTS was added to each well to achieve final concentrations of 0.04 mg/ml. PAC proliferation was quantified after 4 hours by densitometric analysis of MTS tetrazolium compound. Optical density was recorded with a microplate reader at 490 nm. Readings were corrected for background optical density by subtracting the readings from medium/MTS incubated at the same time in the absence of cells.

### Senescence-associated β-Galactosidase assay

Senescence-associated β-Galactosidase staining was performed using a commercial kit (Cell signalling technology, Danvers, MA).

### Capillary-like tube formation on Matrigel

The angiogenic activity of MAECs and HUVECs was determined using a Matrigel tube formation assay. Briefly, after transfection, cells were plated at a density of 20 000 cells/well in 96-well plates precoated with 50 μl of growth factor reduced Matrigel Matrix (Becton Dickinson Labware, Bedford, MA) and cultured at 37^o^C for 6h with 50 ng/mL of VEGF (R&D systems, Minneapolis, MN). Capillary-like tubes were observed under a light microscope. Images were obtained at a 50x magnification and all tubes were counted.

### Scratch assay

Measurement of cell migration was performed using an adapted scratch assay in confluent MAECs and HUVECs. The cells were transfected and grown to near confluence in 24-well plates. Mechanical disruption of the monolayer was realized by scraping with a pipette tip. Migration was assessed using an inverted microscope at a magnification of 200x by an investigator blinded to the experimental conditions. Three fields per well were evaluated and all experiments were performed in duplicate.

### Murine ischemic hindlimb model

The protocol was approved by the Comité Institutionnel de Protection des Animaux (CIPA) of the Centre Hospitalier de l'Université de Montréal (CHUM). 6 to 8 week-old (young) and 15 to 24 month-old (old) C57Bl/6 mice were purchased from Charles River (St-Constant, Qc, Canada). A total of 15 young mice and 23 old mice underwent surgical ischemia. 6 old mice died after surgery, so that 17 old mice were available for the experiments. Unilateral hindlimb ischemia was surgically induced after anesthesia with 2% isoflurane as previously described [37]. The femoral artery was removed from the lateral circumflex branch down to the popliteal artery. Buprenorphine SR (1mg/kg) and bupivacaïne 0.25% were administered during surgery. Mice were injected intramuscularly with 5 mg/kg of in vivo ready mirVana miRNA mimic mmu-miR130a or mirVana miRNA mimic negative control #1 (Life technologies). This dose was chosen based on preliminary experiments showing optimal transfection efficiency in muscles [37]. miRNAs were administered in a solution of Max suppressor RNA-LANCEr II (Bioo Scientific, Austin, TX) according to the manufacturer’s recommendations. Ischemic damages were evaluated using a scale from 1 (no damage) to 4 (loss of toes) and ambulatory impairment was evaluated using a scale from 1 (normal walking) to 4 (walking with the leg dragging behind). The mice were killed at predetermined arbitrary time points after surgery with an overdose of sodium pentobarbital.

### Monitoring of blood flow

Hindlimb blood flow was monitored with a laser Doppler perfusion imager (LDPI) system (Moor Instrument Ltd., Axminster, UK) after anaesthesia with a ketamine–dexmedetomidine solution (50 mg/kg and 0.5 mg/kg, IP). Measurements were performed in the supine position with the legs lying flat on the surgical carpet. The legs were put at a 90-degree angle at the heel to achieve similar degrees of rotation. The region of interest was the distal part of the leg (including the foot), and data acquisition was performed from the knee down to the tip of the toes. Laser Doppler analyses were performed by a single observer blinded to the treatment group at days 0, 3, 7 and 21 after surgery. After LDPI measurements, dexmedetomidine was antagonized with a solution of atipamezole (1 mg/kg, SC). To account for variables such as ambient light and temperature, the results are expressed as the ratio of perfusion in the left (ischemic) versus right (non-ischemic) hindlimb.

### CD31 immunohistochemistry

Whole ischemic hindlimbs were harvested 21 days after surgery and immediately fixed in Tissufix (Chaptec, Montreal, QC, Canada) overnight. After bones were carefully removed, 3-mm-thick tissue transverse sections of the hindlimbs were cut at the level of the gastrocnemius muscle and paraffin-embedded so that the whole leg could be analysed on each section. Identification of endothelial cells was performed by immunohistochemistry for CD31 with a rat monoclonal antibody directed against mouse CD31 (BD Pharmingen, San Diego, CA, USA). Capillaries were counted by a single observer blinded to the treatment regimen at a 200x magnification. Results were expressed as capillaries per field.

### Western blot analysis

Protein levels were analysed by Western blots in ischemic muscles homogenates. For total protein extraction, isolated muscles from whole hindlimbs were rinsed in PBS to remove excess blood, snap-frozen in liquid nitrogen and stored at -80°C until use. Whole-cell protein extracts were obtained after homogenization of ischemic muscles of the different groups of mice in ice-cold RIPA buffer (pH = 8) containing 50 mM Tris–HCl, 150 mM NaCl, 5 mM EDTA, 1% Triton X-100, 0.5% sodium deoxycholate, 0.1% SDS with a cocktail of proteases and phosphatase inhibitors (MiniComplete, PhosphoStop and PMSF, Roche, Risch-Rotkreuz, Switzerland). Samples were separated on an SDS-polyacrylamide gel and electroblotted on PVDF membranes. Non-specific binding sites were blocked with 5% skim milk powder in TBS-T (50 mM Tris–HCl, 140 mM NaCl, 0.05% Tween-20) for 1 hour. The membranes were probed overnight at 4°C with the following antibodies: MEOX2 (1:10 000; Abcam, Cambridge, England), HOXA5 (1:1000; Abcam) or β-actin (1:10 000, Santa Cruz Biotechnology). Membranes were then washed with TBS-T and incubated with anti-rabbit or anti-mouse secondary antibodies conjugated with HRP for 1 hr and washed with TBS-T. Specific proteins were detected by chemiluminescent reaction (BioRad, Hercules, CA), imaged using a ChemiDoc imager (BioRad) and analysed with the Image Lab Software (BioRad). The results are expressed as density values normalized to the loading control (β-actin).

### Plasma NO release

NO production was determined indirectly using a commercial kit that measures the concentration of the stable end products nitrate and nitrite based on the Griess reaction (R&D systems).

### PAC isolation and characterization

7 days after hindlimb ischemia, mouse bone marrow mononuclear cells were isolated from the femora and tibiae by flushing the bone marrow cavities using culture medium (M200 supplemented with 18% FBS, LSGS and 1% penicillin-Streptomycin), and kept on fibronectin (Sigma, St. Louis, MO)-coated plates. After 4 days in culture, non-adherent cells were removed by thorough washing with PBS. Adherent cells were stained with DAPI (0.5 mg/ml; Life Technologies), 1,10-dictadecyl-3,3,30,30 acetylated low-density lipoprotein (DiI-acLDL, 2.5 mg/ml for 1 h, Life Technologies) and FITC-labelled lectin BS-1 (Bandeiraea simplicifolia, 10 mg/ml for 1 h, Sigma, St-Louis, MO). Pro-angiogenic cells (PACs), also known as ‘Early outgrowth EPCs’ can express endothelial markers but also myeloid markers such as CD45 and CD14, attesting for a probable monocytic origin. In our experiments, spindle-shaped cells were observed, and the vast majority of adherent cells (95%) were found to be double-positive for the uptake of DiI-labeled acetylated LDL and binding of FITC-labeled lectin. Characterization of our mouse bone marrow PAC population by FACS analysis indicates that 83% of adherent cells express CD45, 57% CD14, 18% CXCR4, 9% CD31 and 8% Sca-1. These values are similar to previous studies in mice and consistent with a probable monocytic origin. We also found that these cells can migrate in response to VEGF stimulation and are capable of incorporating into a network of tubular-like structures when cocultured with mature endothelial cells. On the basis of these morphological and functional characteristics and in line with previous studies, these cells were characterized and referred to in the manuscript as PACs.

### Incorporation of PACs into HUVECs tubules

PACs (4000) labeled 1 hour with DiI-acLDL were co-plated with HUVECs (20 000) in 96-well plates precoated with 50μl of growth factor reduced Matrigel Matrix (BD Biosciences, San Diego, CA, USA) and cultured at 37ºC for 6h with 50 ng/mL of VEGF. Tubular-like structures were photographed and the number of incorporated PACs was determined in 6 random fields. A tube was defined as a straight cellular segment connecting two cell masses (nodes). No difference in the total number of tubes or in tube length was observed between the different groups (data not shown). The data are presented as number of incorporated PACs/tube.

### PACs migration assay

PAC migration was assessed using a modified Boyden chamber assay; 15 000 cells in growth factor deprived medium were added to the upper chamber of a transwell insert (pore size 8 μm; Corning, Corning, NY, USA) coated with 0.1% gelatin. The inserts were placed in a 24-well plate containing medium 200 with 50 ng/ml VEGF. After incubation for 6 hours at 37°C, the cells which did not migrate were removed by wiping the upper surface with an absorbent tip. Migrated cells were fixed for 10 min with 3.7% formaldehyde and stained 10 min with haematoxylin. The number of cells that had migrated was counted in three different representative high-power (200x) fields per insert. All experiments were performed in duplicate.

### Statistical analysis

All results are mean ± SEM. Statistical significance was evaluated by unpaired T test or ANOVA followed by a Newman Keuls post hoc test. For the Laser Doppler measurements, a 2-way repeated measures ANOVA was used. A value of P<0.05 was interpreted to denote statistical significance.
